# Association between antimicrobial drug class for treatment and retreatment of bovine respiratory disease (BRD) and frequency of resistant BRD pathogen isolation from veterinary diagnostic laboratory samples

**DOI:** 10.1371/journal.pone.0219104

**Published:** 2019-12-13

**Authors:** Johann F. Coetzee, Drew R. Magstadt, Pritam K. Sidhu, Lendie Follett, Adlai M. Schuler, Adam C. Krull, Vickie L. Cooper, Terry J. Engelken, Michael D. Kleinhenz, Annette M. O’Connor

**Affiliations:** 1 Department of Veterinary Diagnostic and Production Animal Medicine, College of Veterinary Medicine, Iowa State University, Ames, IA, United States of America; 2 Department of Anatomy and Physiology, College of Veterinary Medicine, Kansas State University, Manhattan, KS, United States of America; 3 Department of Information Management and Business Analytics, College of Business and Public Administration, Drake University, Des Moines, IA, United States of America; 4 Department of Clinical Sciences, College of Veterinary Medicine, Kansas State University, Manhattan, KS, United States of America; University of Minnesota, UNITED STATES

## Abstract

Although 90% of BRD relapses are reported to receive retreatment with a different class of antimicrobial, studies examining the impact of antimicrobial selection (i.e. bactericidal or bacteriostatic) on retreatment outcomes and the emergence of antimicrobial resistance (AMR) are deficient in the published literature. This survey was conducted to determine the association between antimicrobial class selection for treatment and retreatment of BRD relapses on antimicrobial susceptibility of *Mannheimia haemolytica*, *Pasteurella multocida*, and *Histophilus somni*. Pathogens were isolated from samples submitted to the Iowa State University Veterinary Diagnostic Laboratory from January 2013 to December 2015. A total of 781 isolates with corresponding animal case histories, including treatment protocols, were included in the analysis. Original susceptibility testing of these isolates for ceftiofur, danofloxacin, enrofloxacin, florfenicol, oxytetracycline, spectinomycin, tilmicosin, and tulathromycin was performed using Clinical and Laboratory Standards Institute guidelines. Data were analyzed using a Bayesian approach to evaluate whether retreatment with antimicrobials of different mechanistic classes (bactericidal or bacteriostatic) increased the probability of resistant BRD pathogen isolation in calves. The posterior distribution we calculated suggests that an increased number of treatments is associated with a greater probability of isolates resistant to at least one antimicrobial. Furthermore, the frequency of resistant BRD bacterial isolates was greater with retreatment using antimicrobials of different mechanistic classes than retreatment with the same class. Specifically, treatment protocols using a bacteriostatic drug first followed by retreatment with a bactericidal drug were associated with a higher frequency of resistant BRD pathogen isolation. In particular, first treatment with tulathromycin (bacteriostatic) followed by ceftiofur (bactericidal) was associated with the highest probability of resistant *M*. *haemolytica* among all antimicrobial combinations. These observations suggest that consideration should be given to antimicrobial pharmacodynamics when selecting drugs for retreatment of BRD. However, prospective studies are needed to determine the clinical relevance to antimicrobial stewardship programs in livestock production systems.

## Introduction

Bovine respiratory disease (BRD) is one of the most important diseases facing the beef cattle industry [1]. Annual economic losses due to BRD are estimated to approach $1 billion in the United States alone [1,2]. Treatment and control of BRD are currently predicated on administration of antimicrobial therapy directed toward the primary bacterial pathogens *Mannheimia haemolytica*, *Pasteurella multocida*, and *Histophilus somni*. Antimicrobial drugs are broadly classified into two groups, namely those that inhibit growth of the organism (i.e., bacteriostatic) and those that kill the organism (i.e., bactericidal). The National Animal Health Monitoring System Feedlot 2011 study reported that 21.2 ± 2.0% (mean ± standard error, SE) of cattle in feedlots were administered antimicrobials to control an expected outbreak of BRD, and approximately 15% of feedlot cattle required a second antimicrobial treatment for the disease [3,4,5]. Although approximately 90% of cases with BRD relapse were reported to receive retreatment with a different antimicrobial mechanistic class [5], studies examining the impact of antimicrobial drug class on retreatment outcomes and the emergence of antimicrobial resistance (AMR) are scarce in the published literature. Knowledge of the impact of antimicrobial drug selection on AMR emergence is needed to develop judicious use guidelines that preserve antimicrobial efficacy and advance antimicrobial stewardship.

Minimum inhibitory concentration (MIC) data obtained from samples submitted to veterinary diagnostic laboratories (VDLs) reflect antimicrobial susceptibility and are commonly used to describe AMR changes in livestock populations [6,7,8]. A retrospective study of *M*. *haemolytica*, recovered from lung samples submitted to the Kansas State University VDL between 2009 and 2011, reported a 7-fold increase in the number of isolates resistant to five or more antimicrobials over a 3-year period [9]. However, the association between antimicrobial treatment and the recovery of a resistant *M*. *haemolytica* isolate could not be evaluated because individual animal treatment histories were not reported. Recently, our group reported an association between treatment history and antimicrobial sensitivity results from bacterial isolates obtained from BRD cases submitted to the Iowa State University VDL (ISU-VDL) from 2013–2015 [10]. Bacterial isolates from cattle that received antimicrobial treatment showed a higher incidence of antimicrobial resistance than isolates from untreated cattle. Furthermore, the percentage of resistant isolates increased with the number of antimicrobial treatments. However, the relationships between the antimicrobial drug class selected for initial treatment and retreatment as well as the frequency of AMR pathogen isolation were not investigated.

It was revealed more than 50 years ago that an overall reduction in antimicrobial efficacy occurs when antimicrobials that cause target organism death (i.e., bactericidal agents) are used in combination with antimicrobials that only inhibit bacterial replication (i.e., bacteriostatic agents). Bacteriostatic antimicrobials inhibit bacterial growth without killing the organism (i.e., bacterial growth is arrested in the stationary phase). Bactericidal antimicrobial agents are more active on actively multiplying bacteria, resulting in cell death. It is therefore hypothesized that in case of bacteriostatic-bactericidal combination, the growth inhibition induced by a bacteriostatic agent may result in an overall reduction of efficacy (reduced growth and killing of bacteria) of the bactericidal agent [11,12]. The resulting drug antagonism is associated with poorer clinical outcomes [13,14]. These findings suggest that the choice of antimicrobial drug class (i.e., bactericidal or bacteriostatic) in cases of relapse and retreatment may be a critical control point for mitigating AMR in beef production systems.

The objectives of this study were to use a Bayesian approach to 1) obtain the posterior distribution of the resistance patterns for the number of treatments (1, 2, 3, or 4+) administered to cases submitted to the ISU-VDL; and 2) test the hypothesis that antimicrobial resistant BRD pathogens are recovered more frequently from calves that received second-line treatment from a different antimicrobial class than from calves that received second-line treatment from the same antimicrobial class.

## Materials and methods

### Study design

This cross-sectional study used data collected from the electronic and paper laboratory records of the ISU-VDL from January 1, 2013 to December 2, 2015, including the original documents, which were used to extract the relevant antimicrobial treatment information. The data were retrieved in 2016.

### Settings

The 1,251 isolates available for analysis were submitted to the ISU-VDL by referring veterinarians from 24 states. The majority of isolates were from Iowa (778), Minnesota (80), and South Dakota (49). Most isolates were obtained from animals housed in feedlots (498), confinement operations (268), or pastures (162). The demographic information from the sample submissions is summarized in S1 and S2 Tables.

### Cases and case isolates

Bacterial isolate data and the corresponding case history information were included in the study upon meeting the following criteria: 1) the submitted samples were from a bovine field case (research cases were excluded); 2) *M*. *haemolytica*, *P*. *multocida*, or *H*. *somni* were isolated via routine culture; 3) the sample that yielded the isolate was from the lower respiratory tract (lung, pleural surface, bronchoalveolar lavage fluid); 4) MIC testing results were available; 5) the submission form stated a history of respiratory disease and/or evidence of pneumonia was described in autopsy findings or upon histological evaluation of lung tissue; and 6) the submitting veterinarian provided a treatment history that included either the generic or trade name of the antimicrobials used in the treatment of the case prior to sample submission.

### Study size

No *a priori* sample size was determined because the study was intended to be cross-sectional and hypothesis-generating. Therefore, sample size was determined solely by the number of eligible isolates available during the study period.

### Variables and data sources

The outcome of interest was the Clinical and Laboratory Standards Institute (CLSI)-validated interpretive category based on MIC.

Susceptibility testing was performed according to standard laboratory methods based on CLSI recommendations [15]. Briefly, the selected culture was grown overnight and a broth dilution was inoculated on a standard 96-well susceptibility plate (BOPO6F, Thermo Scientific, Oakwood Village, OH, USA) using an automated inoculation system (Sensititre AIM, Thermo Scientific). Susceptibility plates were read using a manual system (Sensititre Vizion System, Thermo Scientific) following 18–24 h incubation at 37°C.

Not all antimicrobial compounds included on the standard susceptibility plate have CLSI-validated interpretive breakpoints; therefore, only antimicrobials with CLSI-approved breakpoints [2] for respiratory disease caused by *M*. *haemolytica*, *P*.*multocida* or *H*.*somni* were included in this study (S3 Table). The antimicrobials included in this study were ceftiofur, danofloxacin, enrofloxacin, florfenicol, oxytetracycline, spectinomycin, tilmicosin, and tulathromycin. Established CLSI-validated breakpoints are not available for tilmicosin against *P*. *multocida* or tilmicosin and danofloxacin against *H*. *somni* in BRD; therefore, these antimicrobials were included in this study using the CLSI-validated breakpoints for *M*. *haemolytica*.

Treatment history was recorded in the paper submission forms by the referring veterinarian. Information regarding the number of antimicrobial treatments, specific antimicrobials used and non-antimicrobial treatments was manually extracted from these records by one investigator (AS). Isolates from submissions that explicitly stated no usage of antimicrobial drugs were assigned the treatment history classification of none (“0”). Isolates from cases in which information regarding antimicrobial treatments was unclear (e.g., “many” or “everything”) or not given were classified as “unknown.” Isolates from cases with treatment histories indicating the use of four or more antimicrobials were classified as “4+.”

Trade names were converted to generic drug names to determine the antimicrobial drug class (bacteriostatic or bactericidal) and the sequence of class administration for first- and second-line treatments. Drug class was assigned based on the established *in vitro* pharmacodynamics of the antimicrobial agent as summarized in **Table 1.**

**Table 1:** Classification of antimicrobial drugs on the basis of antimicrobial activity.

Bactericidal	Bacteriostatic
Ceftiofur	Chlortetracycline
Danofloxacin	Florfenicol
Enrofloxacin	Gamithromycin
Penicillin	Oxytetracycline
	Spectinomycin
	Sulfadimethoxine
	Tildipirosin
	Tilmicosin
	Tulathromycin
	Tylosin

Data on potential confounders or effect modifiers extracted from the submission form, including breed, sex, facility type, clinical signs, necropsy findings, vaccination status, and weights, were recorded (**S1 and S2 Tables**). Finalized case report information, such as microscopic evidence of pneumonia, also was noted. Case information was classified as “unknown” if the information was not supplied or unclear. After each eligible record was identified, the submission forms for each case were individually reviewed by a single researcher (AS). Antimicrobial treatments were grouped as -cidal or -static based on expected antimicrobial pharmacodynamics.

### Variable transformations

Due to sparse data for cases receiving multiple treatments, we arbitrarily chose to group together animals that received more than three treatments (4+). Animals with unknown treatment histories were excluded from the analysis.

For the subset of animals receiving just two treatments, we created two categorical variables. One categorical variable grouped the data into two levels: “same” to designate animals that received first- and second-line treatment from the same drug class (i.e., either bacteriostatic and bacteriostatic or bactericidal and bactericidal) and “different” to designate animals that received first- and second-line treatment from different drug classes (i.e., either bacteriostatic followed by bactericidal or bactericidal followed by bacteriostatic). We also created a four-level categorical variable to capture all possible combinations (4 levels: bacteriostatic followed by bactericidal, bacteriostatic followed by bacteriostatic, bactericidal followed by bacteriostatic, and bactericidal followed by bactericidal).

### Statistical analysis

Initial analysis included descriptive statistics to illustrate the distribution of the number of treatments cross tabulated with the number of antimicrobials of which the isolate was classified as resistant. The number of missing values also was determined. We created heat maps to show the pairwise interactions of antimicrobial treatment combinations associated with the isolation of resistant *M*. *haemolytica*. This analysis was not conducted for *P*. *multocida* and *H*. *somni* due to the smaller number of isolates for each treatment combination that were available for analysis.

The approach for addressing our two objectives was to conduct a Bayesian analysis for isolates of *M*. *haemolytica*, *P*. *multocida*, and *H*. *somni* using a finite mixture model based on a zero-inflated beta-binomial model. The open source software R was used to conduct this analysis. For both objectives, we let *y*_*ij*_ represent the number of resistant organisms, where *i* represents the level of the explanatory variable treatment and *j* represents the organism. We assume the observations are conditionally independent. We write the model as follows:

$${(y}_{ij}|\gamma_{i,}z_{ij}=1)∼Binomial({18,\gamma }_{i})$$

$${(P\;(y}_{ij}=0|\gamma_{i,}z_{ij}=0)=1$$

where *z*_*ij*_ represents the category (i.e., antimicrobial drug class) and n = 18 represents the number of possible antimicrobials. Thus, the probability density function of *y*_*ij*_ is:

((18yij)γiyij(1−γi)18−yij)zij(I(yij=0))1−zij

we allow the category indicator, *z*_*ij*_, to also be conditionally independent with the following distributional assumption:

$$z_{ij}|p_{i}∼Bernouli\;(p_{i})$$

*Rho* (*p*_*i*_) and *gamma* (*γ*_*i*_) are assumed to be independent with priors specified as follows:

$$\gamma_{i}∼Beta\;(a,b)$$

$$p_{i}∼Beta\;(a,b)$$

where *a* = 1 and *b* = 1.

For Objective 1, *i* in the model referred to the number of treatments reported by the submitter (i.e., *i* had five levels: 0, 1, 2, 3, and 4+ treatments). For Objective 2, two models were created for the subset of animals that received two treatments. For the first model (Objective 2 Model 1), γ_i_ referred to the two-level sequence of treatments reported by the submitter (i.e., *i* had two levels: same and different). For the second model (Objective 2 Model 2), γ_i_ referred to the four-level sequence of treatments reported by the submitter (i.e., *i* had four levels: bactericidal-bactericidal, bactericidal-bacteriostatic, bacteriostatic-bactericidal, and bacteriostatic-bacteriostatic). We sampled from the joint posterior distribution of γ_i_ and ρ_i_ implied by the model using a Metropolis random walk Markov chain Monte Carlo (MCMC) approach.

One output from each model was the posterior distribution of ρ_*i*_ based on each *i*^th^ level of the explanatory variable; i.e., ρ_i_ is the probability that an animal in group *i* comes from the binomial distribution. We use this posterior distribution to make inferences about the data. For example, we are interested in the probability that an organism is resistant to at least one antimicrobial, which is given by ρ_i_* (probability binomial [18, γ_i_] random variable > 0). We are also interested in whether this probability is associated with either the number of times an animal is treated or the treatment sequence. The other output was the posterior distribution of γ_i_ based on each *i*^*th*^ level of the explanatory variable. Posterior γ distributions that are shifted to the right indicate an isolate that is resistant to a higher number of antimicrobials.

To examine the relationship between the probability of at least one resistant test result and the number of treatments, we determined the posterior distribution of *p*_*i+1*_ > *p*_*i*_, i.e., how often the probability of having at least one resistant test was higher for *i + 1* treatments compared to *i* treatments.

After creating the models, we assessed several discrepancy measures, including the number of zeros in the posterior distribution of y* and the number of extreme values. This first reflects the inflation of zeros in the observed distribution and is mainly informed by the posterior distribution of ρ_*i*._ The second measure assesses the distribution of resistant organisms, given that they are resistant, and is mainly informed by the posterior distribution of γ_i._ For each posterior distribution of γ_i_ and ρ_i_, we reported the 95% credible interval (95% CI) and also the posterior probability that γ_i_ < γ_≠i_ and ρ_i_ <ρ_≠i._ for all possible pairwise comparisons.

### Ancillary analyses and sensitivity analyses

We originally intended to conduct further subgroup analysis based on the particular isolates of *M*. *haemolytica*, *P*. *multocida*, and *H*. *somni*. However, on further examination, we determined that the data were too sparse to warrant further subgrouping. We also were originally interested in the impact of two covariates, simultaneous viral and *Mycoplasma* spp. infection, on the posterior distribution; however, descriptive analysis indicated that this approach was unlikely to be useful. Thus, although we extracted these data, we did not conduct these analyses.

## Results

A total of 1,251 bacterial isolates were available for our analysis, including 540 isolates of *M*. *haemolytica*, 404 isolates of *P*. *multocida*, and 307 isolates of *H*. *somni*. Isolates were obtained from 1,031 individual animals under 989 case submissions by 378 veterinarians. **Table 2** summarizes the numbers of each organism isolated each year over the course of the study.

**Table 2:** Summary of bacterial isolates obtained from submitted samples of animals treated with bacteriostatic/bactericidal antimicrobial agents.

Year	2013			2014			2015			Total
Organisms (culture)	MH	PM	HS	MH	PM	HS	MH	PM	HS	
Isolates from submissions with treatment history	113	56	52	127	90	81	106	94	62	**781**
**Total isolates/year**	**221**			**298**			**262**			
**Isolates from non-treated cases**	28	19	13	27	29	17	25	19	17	**194**
	**60**			**73**			**61**			
**Isolates from first-line bactericidal treatment**										
Ampicillin	0	0	0	1	0	0	0	2	0	3
Ceftiofur	14	1	5	15	10	11	13	8	4	81
Danofloxacin	0	1	0	1	1	0	0	0	0	3
Enrofloxacin	12	5	6	10	9	11	13	14	5	85
Penicillin	2	2	0	0	0	0	2	2	1	9
**Total**	**48**			**69**			**64**			**181**
**Isolates from first-line bacteriostatic treatment**										
Chlortetracycline	0	0	0	4	4	1	1	0	1	11
Florfenicol	6	6	7	8	7	9	16	8	8	75
Gamithromycin	2	1	0	9	6	2	5	1	2	28
Oxytetracycline	2	1	1	0	3	1	1	2	1	12
Sulfadimethoxine	2	2	0	0	0	1	1	0	0	6
Tetracycline	0	0	0	5	2	2	0	1	2	12
Tildipirosin	11	3	4	6	5	6	4	8	3	50
Tilmicosin	4	4	4	8	1	5	2	2	0	30
Tulathromycin	29	10	11	33	13	15	24	26	18	179
Tylosin	1	1	1	0	0	0	0	0	0	3
**Total**	**113**			**156**			**137**			**406**

(MH = *M*. *haemolytica*, PM = *P*. *multocida*, HS = *H*. *somni)*.

The data set of 781 out of 1,251 bacterial isolates was used for analysis because 470 isolates did not have treatment information included with the sample submission. The dataset available for Objective 1 included 781 isolates, of which 194 received 0 treatments, 276 received 1 treatment, 211 received 2 treatments, 74 received 3 treatments, 23 received 4 treatments, 2 received 5 treatments, and 1 received 7 treatments. Missing data for this subset are presented in **S1 Table**. A total of 211 isolates were from animals that received only two treatments. These isolates were treated with the same drug class in 97 cases (18 bactericidal-bactericidal and 79 bacteriostatic-bacteriostatic) and 114 were treated with different drug classes (52 bactericidal-bacteriostatic and 62 bacteriostatic-bactericidal).

**Fig 1:**
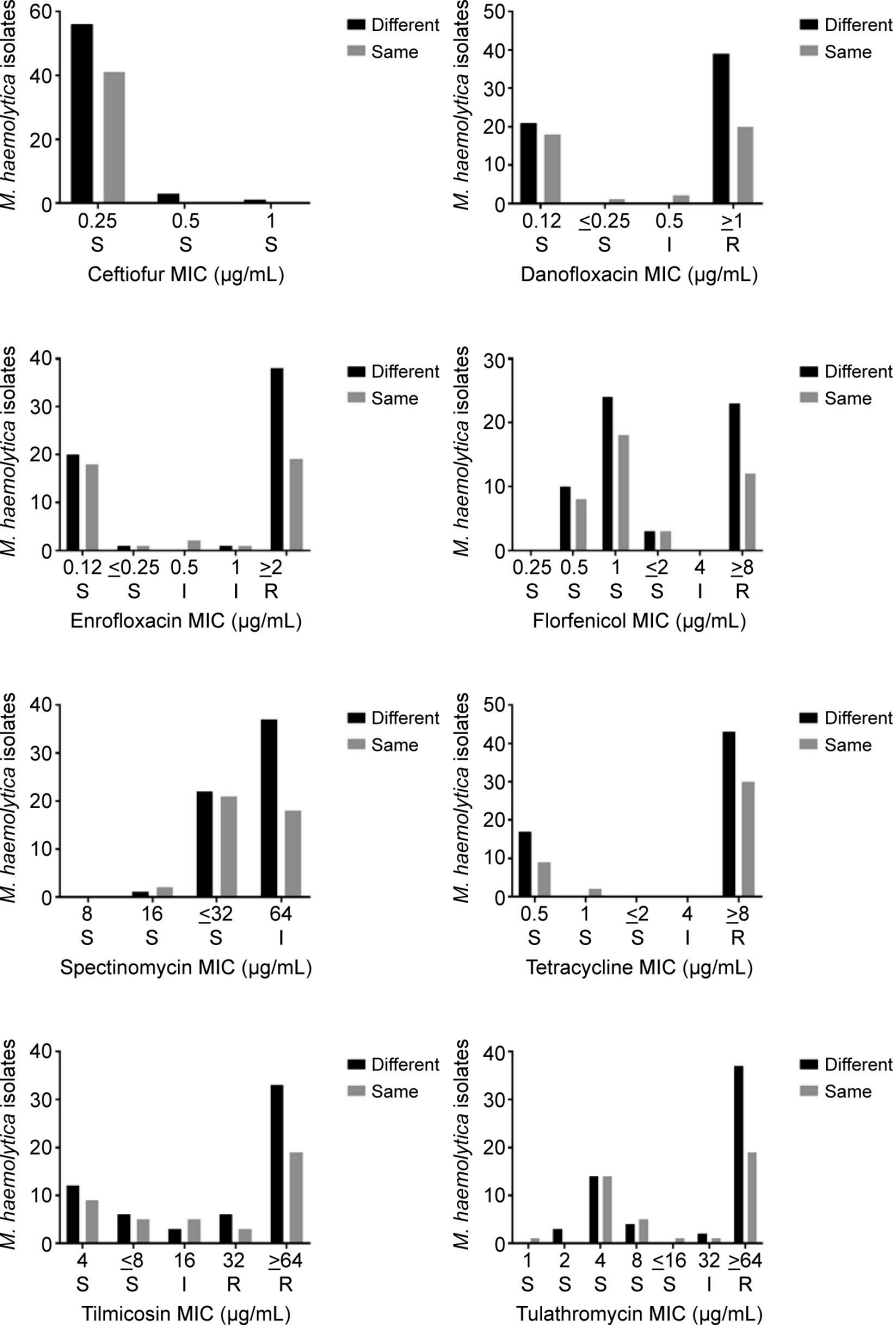
Distribution of minimum inhibitory concentrations (MIC) of antimicrobial agents with CLSI approved breakpoints for *M*. *haemolytica* in cattle receiving the same or different treatments. S = susceptible; I = intermediate; R = resistant. For “same” treatments, the first and second treatments were either bactericidal drugs or bacteriostatic drugs. For “different” treatments, the first treatment was bactericidal and second was bacteriostatic or *vice versa*.

The observed antimicrobial susceptibility profiles for *M*. *haemolytica* based on MIC data of cattle administered either the “same” (first and second treatment were both either bactericidal drugs, or bacteriostatic drugs) or “different” (first treatment was bactericidal and second was bacteriostatic or *vice versa*) antimicrobial treatment are presented in **Fig 1.** A similar examination of the data was not conducted for *P*. *multocida* and *H*. *somni* because there were an insufficient number of isolates for this to be meaningful.

**Fig 2:**
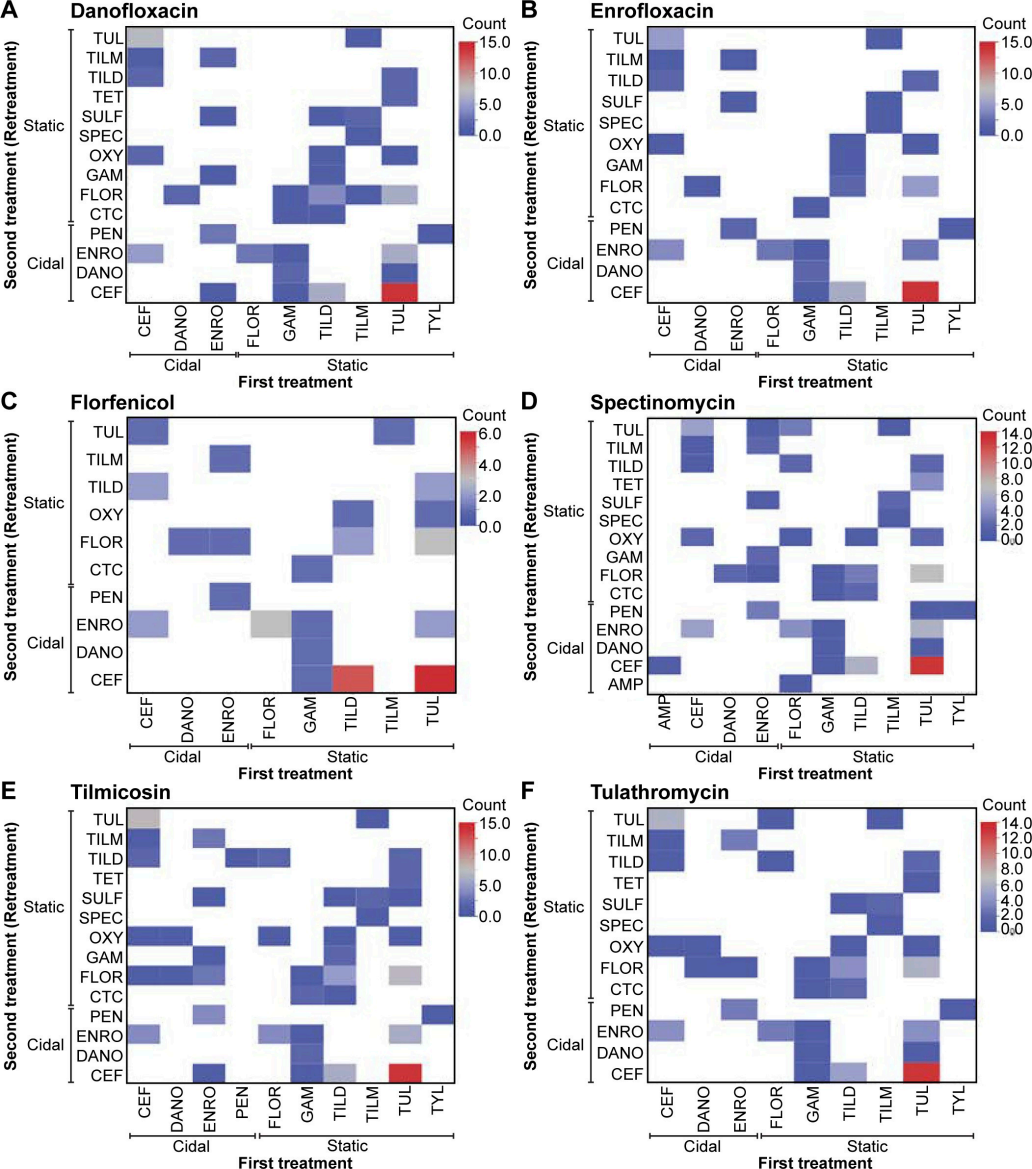
Heat maps showing pairwise interactions of antimicrobial treatment combinations associated with the isolation of resistant *M*. *haemolytica* organisms. The effect of treatment with ceftiofur (CEF), danofloxacin (DANO), enrofloxacin (ENRO), florfenicol (FLOR), gamithromycin (GAM), oxytetracycline (OXY), penicillin (PEN), spectinomycin (SPEC), sulfadimethoxine (SULF), tetracycline (TET), tildipirosin (TILD), tilmicosin (TILM), tulathromycin (TUL), and tylosin (TYL) as either first (X-axis) or second (Y-axis) treatment on the frequency of isolating *M*. *haemolytica* organisms resistant to danofloxacin (A), enrofloxacin (B), florfenicol (C), spectinomycin (D), tilmicosin (E) and tulathromycin (F) was examined using CLSI interpretive criteria. White indicates no observation of antimicrobial resistance with that specific combination.

Antimicrobial treatments were grouped based on their anticipated impact on bacterial growth *in vitro*, i.e., bactericidal (“cidal”) or bacteriostatic (“static”). We created a heat map to illustrate the impact of specific pairs of combinations of first and second antimicrobial treatments on the number of isolates identified as resistant against the listed antimicrobials with CLSI breakpoints (**Fig 2**). Red indicates the observed maximum number of resistant isolates and white (i.e., blank) represents no observation of antimicrobial resistance for a specific antimicrobial combination **(Fig 2).** A similar examination of the data was not conducted for *P*. *multocida* and *H*. *somni* because there were an insufficient number of isolates for this to be meaningful.

**Fig 3:**
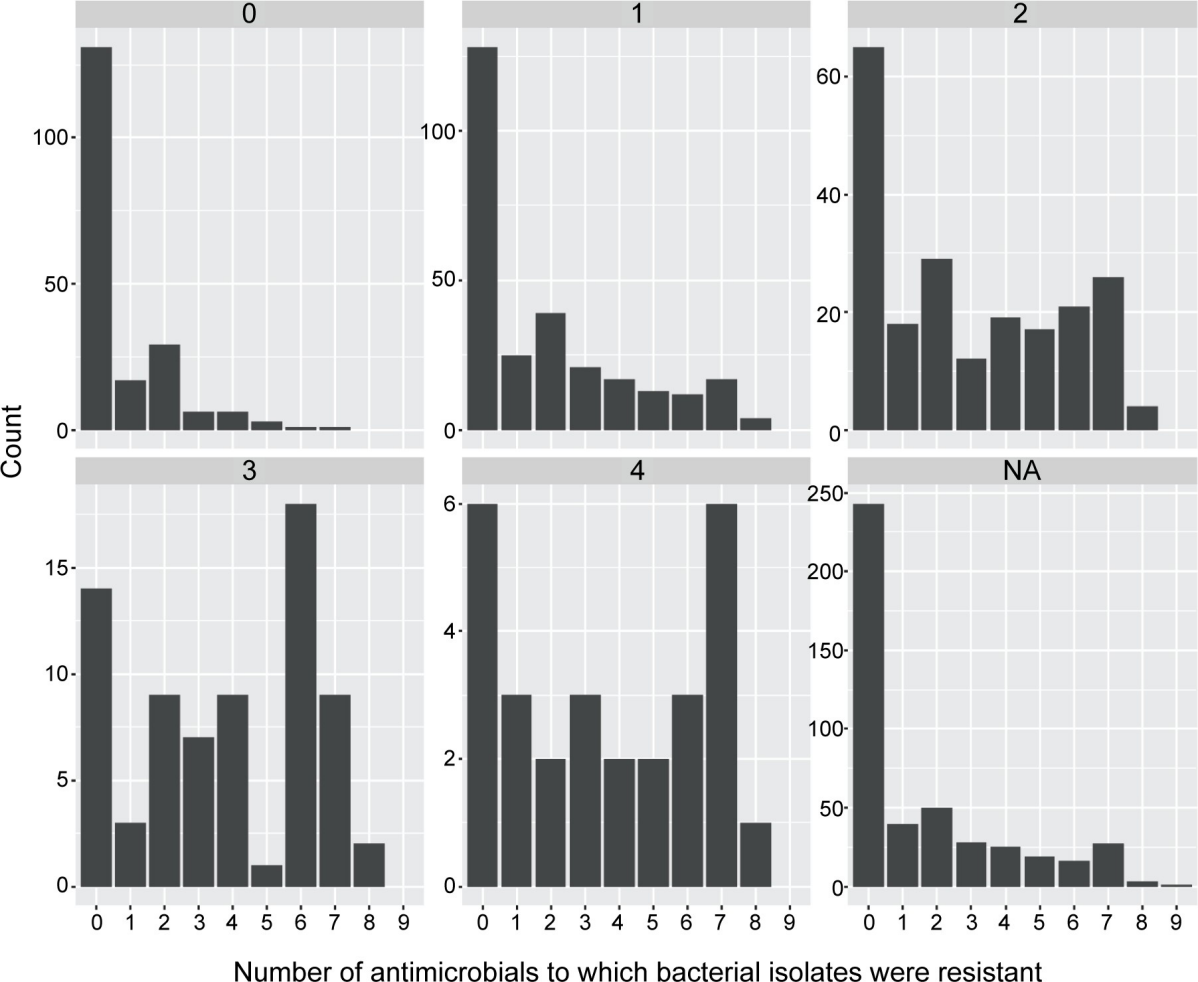
Observed frequency distribution of antimicrobial resistant isolates based on CLSI breakpoints for animals receiving antimicrobials for BRD. 0 = no treatment, 1 = 1 treatment, 2 = 2 treatments, 3 = 3 treatments, 4 = 4 or more treatments, and NA = missing information.

The distribution of AMR in bacterial isolates demonstrated an association between the isolation of an AMR bacteria and the number of treatments used (**Fig 3 and Table 3**). The data indicate that administration of two or more antimicrobial agents to treat BRD in cattle may increase the likelihood of isolating an antimicrobial resistant pathogen (**Fig 3).**

**Table 3:** 95% credible intervals (CIs) for the posterior distributions representing the probability of having at least one resistance result to at least one of the assessed antimicrobials (i.e., ρ) based on CLSI breakpoints stratified by the number of antimicrobials the animal received.

Objective, Model	Percentile		
Objective 1 Model 1: treatment frequency (n = 781)	2.5%	50%	97.5%
0 treatments	0.29	0.36	0.55
1 treatment	0.49	0.55	0.61
2 treatments	0.63	0.69	0.76
3 treatments	0.69	0.80	0.88
4+ treatments	0.61	0.78	0.90
Two treatment sequences (n = 211)			
Same (bactericidal + bactericidal, bacteriostatic + bacteriostatic)	0.61	0.71	0.79
Different (bacteriostatic + bactericidal, bactericidal + bacteriostatic)	0.59	0.69	0.76
Four treatment sequences (n = 211)			
Bactericidal + bactericidal	0.54	0.77	0.92
Bactericidal + bacteriostatic	0.51	0.64	0.76
Bacteriostatic + bacteriostatic	0.59	0.69	0.79
Bacteriostatic + bactericidal	0.60	0.72	0.82

**Fig 4:**
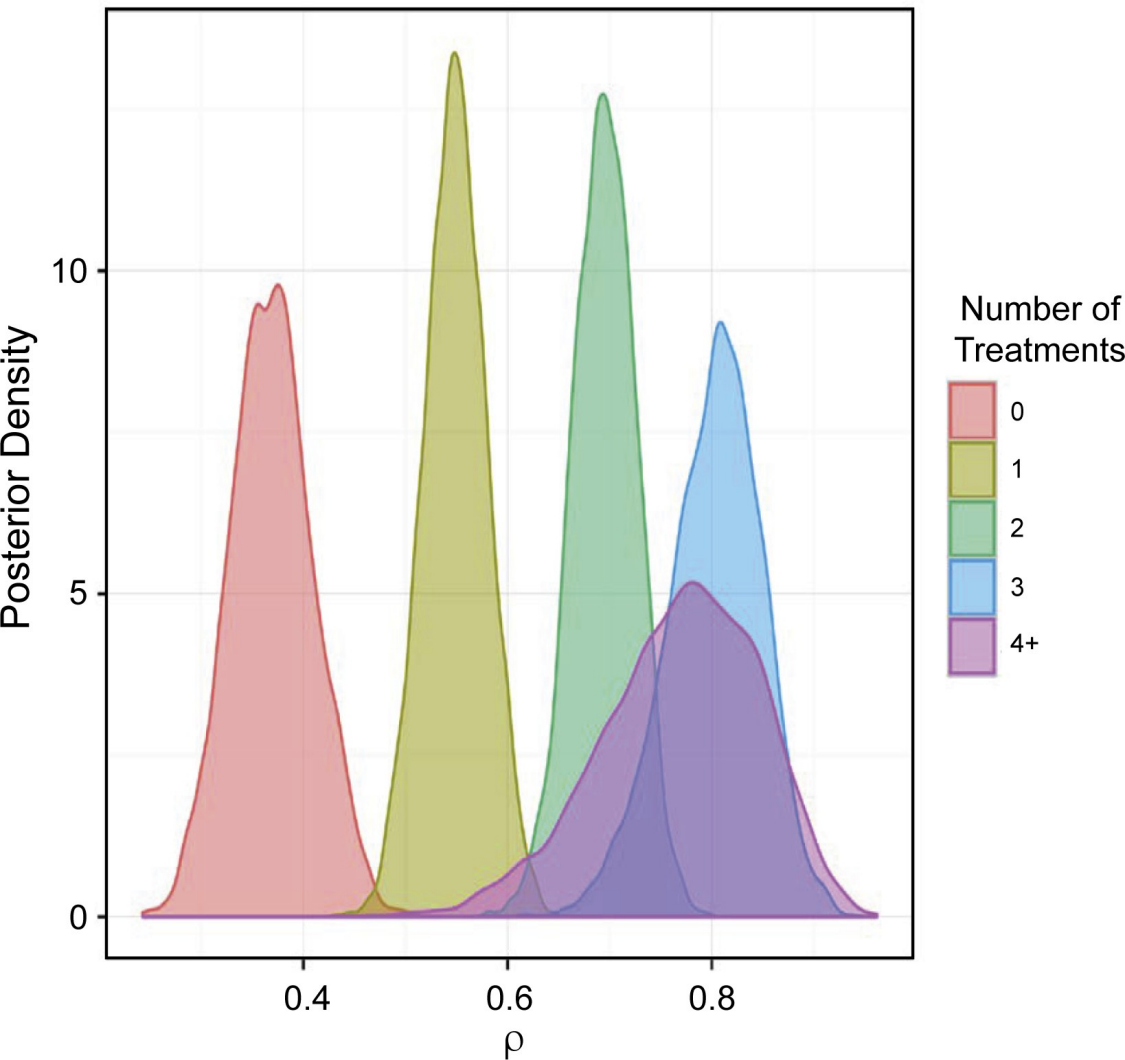
Posterior distribution of the probability that the isolate is resistant to at least one antimicrobial (i.e.,ρ) stratified by treatment frequency. 0 = no treatment, 1 = 1 treatment, 2 = 2 treatments, 3 = 3 treatments, and 4+ = 4 or more treatments.

For Objective 1, the posterior distribution for ρ (i.e., the probability of being resistant to at least one antimicrobial) is provided in **Fig 4.**

The 95% CI for the ρ distribution is provided in **Table 3.** Based on the interpretation of the posterior distributions, the use of antimicrobials was associated with an increased probability of having at least one resistant outcome because the median and 95% CIs shift to the right toward higher probabilities as the number of antimicrobial treatments increased. In addition, there was evidence of an exposure response (i.e., increasing the number of treatments increases the probability of at least one resistant test). The evidence for a response to increasing antimicrobial exposure can be found in **Table 4.**

**Table 4:** Posterior probability that the posterior distribution of *p*_*i+1*_ > *p*_*i*_.

Objective 1 Model 1	0 treatments	1 treatment	2 treatments	3 treatments	4+ treatments
0 treatments	NA	0.99	1	1	1
1 treatment	-	NA	0.99	1	0.99
2 treatments	-		NA	0.95	0.82
3 treatments	-	-		NA	0.40
4+ treatments	-	-	-		NA

*i* represents the number of treatment approaches that differ based on Objective 1 Model 1.

As reported in **Table 4**, ρ increased as the number of reported treatments increased. For example, 40%, 82%, 99%, and 100% of the time, ρ was higher if animals received more than 4 treatments when compared to ρ for 3 treatments, 2 treatments, 1 treatment, or 0 treatments, respectively. When ρ values were entered into the Bernoulli distribution, they translated into a higher prevalence of isolates with at least one resistant outcome.

**Fig 5:**
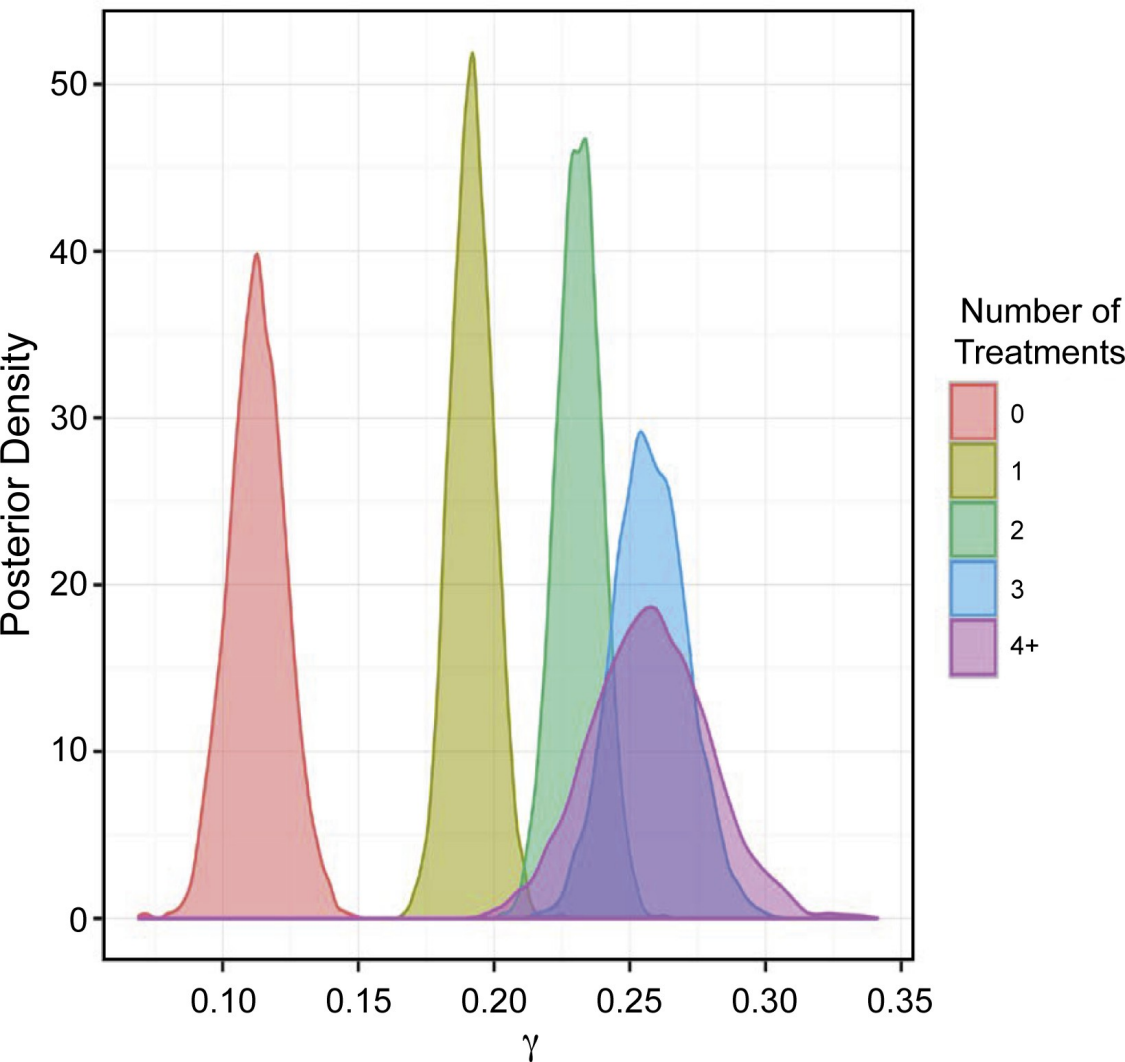
Posterior distributions of the probability that the isolate is resistant to multiple antimicrobials (i.e., γ_i_) stratified by treatment frequency. 0 = no treatment, 1 = 1 treatment, 2 = 2 treatments, 3 = 3 treatments, and 4+ = 4 or more treatments.

The posterior distributions of γ (where γ_i_ = number of resistant tests for each isolate) are shown in **Fig 5** (95% CI in **Table 5**).

**Table 5:** Credibility percentiles for posterior distributions for the number of resistant test results from an isolate (γ_i_).

	Percentile		
Objective 1 Model 1: Treatment frequency	2.5%	50%	97.5%
0 treatments	0.09	0.11	0.13
1 treatment	0.17	0.19	0.21
2 treatments	0.21	0.23	0.25
3 treatments	0.23	0.26	0.28
4+ treatments	0.21	0.25	0.30
Objective 2 Model 1: 2-treatment sequences			
Same (bactericidal + bactericidal, bacteriostatic + bacteriostatic)	0.18	0.20	0.23
Different (bacteriostatic + bactericidal, bactericidal + bacteriostatic)	0.23	0.25	0.28
Objective 2 Model 2: 4-treatment sequences			
Bactericidal + bactericidal,	0.18	0.23	0.29
Bactericidal + bacteriostatic,	0.17	0.21	0.24
Bacteriostatic + bacteriostatic,	0.17	0.19	0.22
Bacteriostatic + bactericidal	0.26	0.28	0.32

Consistent with the results for ρ, there was evidence that increased exposure to antimicrobials resulted in a higher probability of an isolate being resistant to more than one antimicrobial (**Table 6**). However, for the difference between 3 treatments and 4+ treatments, there was only 49% probability (50/50) of one being higher than the other, suggesting a possible threshold or an imprecise estimate of the γ_i_ posterior distribution.

**Table 6:** Posterior probability that γ_i+1_ is greater than γ_i_ where *i* is the number of treatment approaches which differ based on objective 1 model 1 and γ_i_ = number of resistant tests for an isolate.

Objective 1 Model 1: Posterior distribution based on treatment frequency					
i	0 treatments	1 treatments	2 treatments	3 treatments	4+ treatments
0 treatments	NA	1	1	1	1
1 treatment	-	NA	0.99	1	0.99
2 treatments	-		NA	0.95	0.87
3 treatments	-	-		NA	0.49
4+ treatments	-	-	-		NA

*i* is the number of treatments administered based on Objective 1 Model 1.

**Fig 6:**
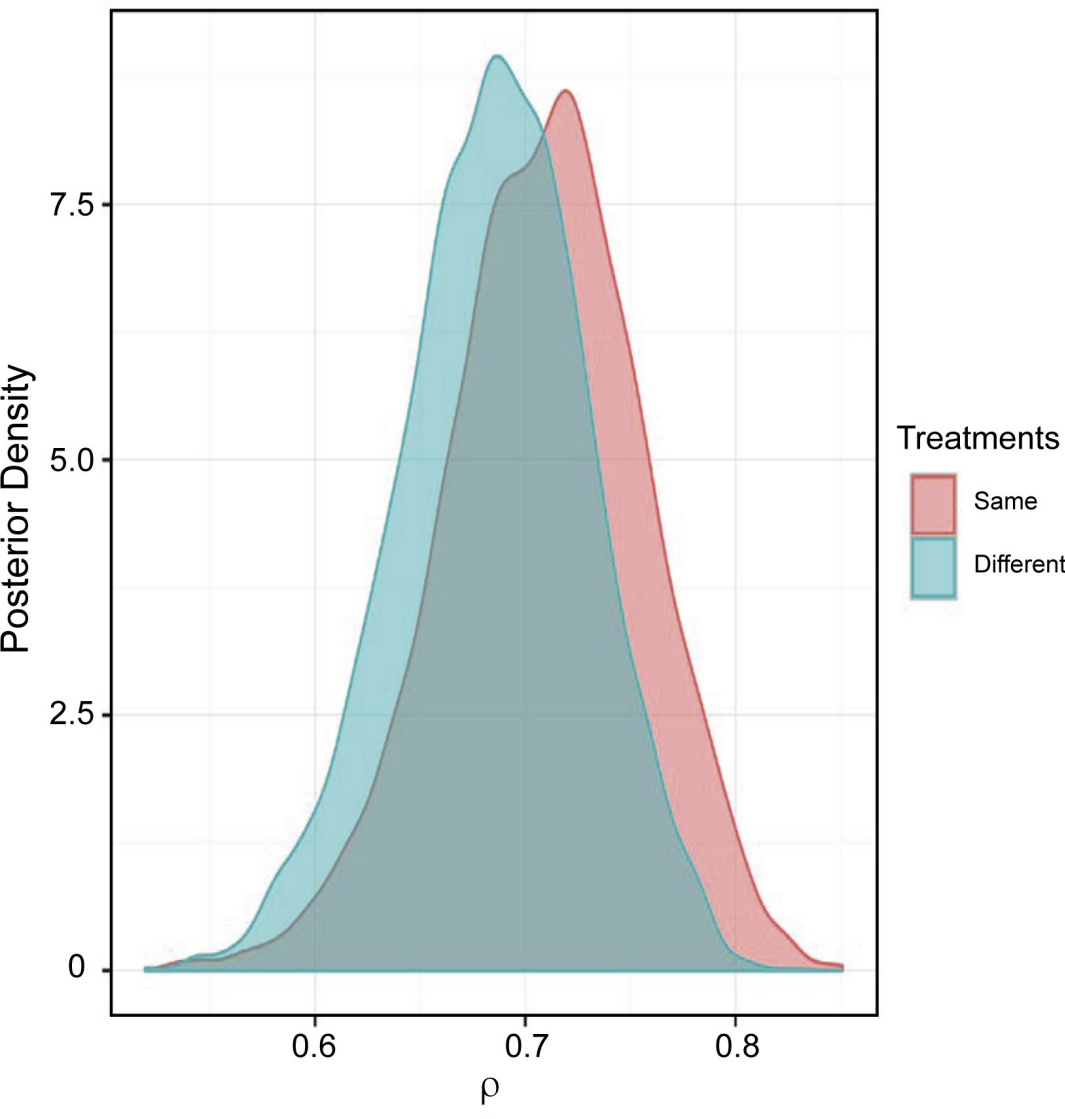
Posterior distribution of the probability that the isolate is resistant to at least one antimicrobial (i.e., ρ_i_) stratified by the expected *in vitro* activity (i.e. bactericidal or bacteriostatic) of first and second treatment. Same = same *in vitro* effect on bacterial growth, i.e., bactericidal followed by bactericidal or bacteriostatic followed by bacteriostatic; or different = different *in vitro* effect on bacterial growth, i.e., bactericidal followed by bacteriostatic or bacteriostatic followed by bactericidal.

Objective 2 examined the development of resistance based on whether the antimicrobial selected for the initial treatment and retreatment would be expected to kill the bacteria *in vitro* (bactericidal) or inhibit the replication of the bacteria *in vitro* (bacteriostatic). As shown in **Fig 6** and **Table 3**, the posterior distribution of ρ (i.e., the probability of the isolate being resistant to at least one antimicrobial) when animals received drugs of the same or different mechanistic classes do not appear to be associated with different distributions.

**Fig 7:**
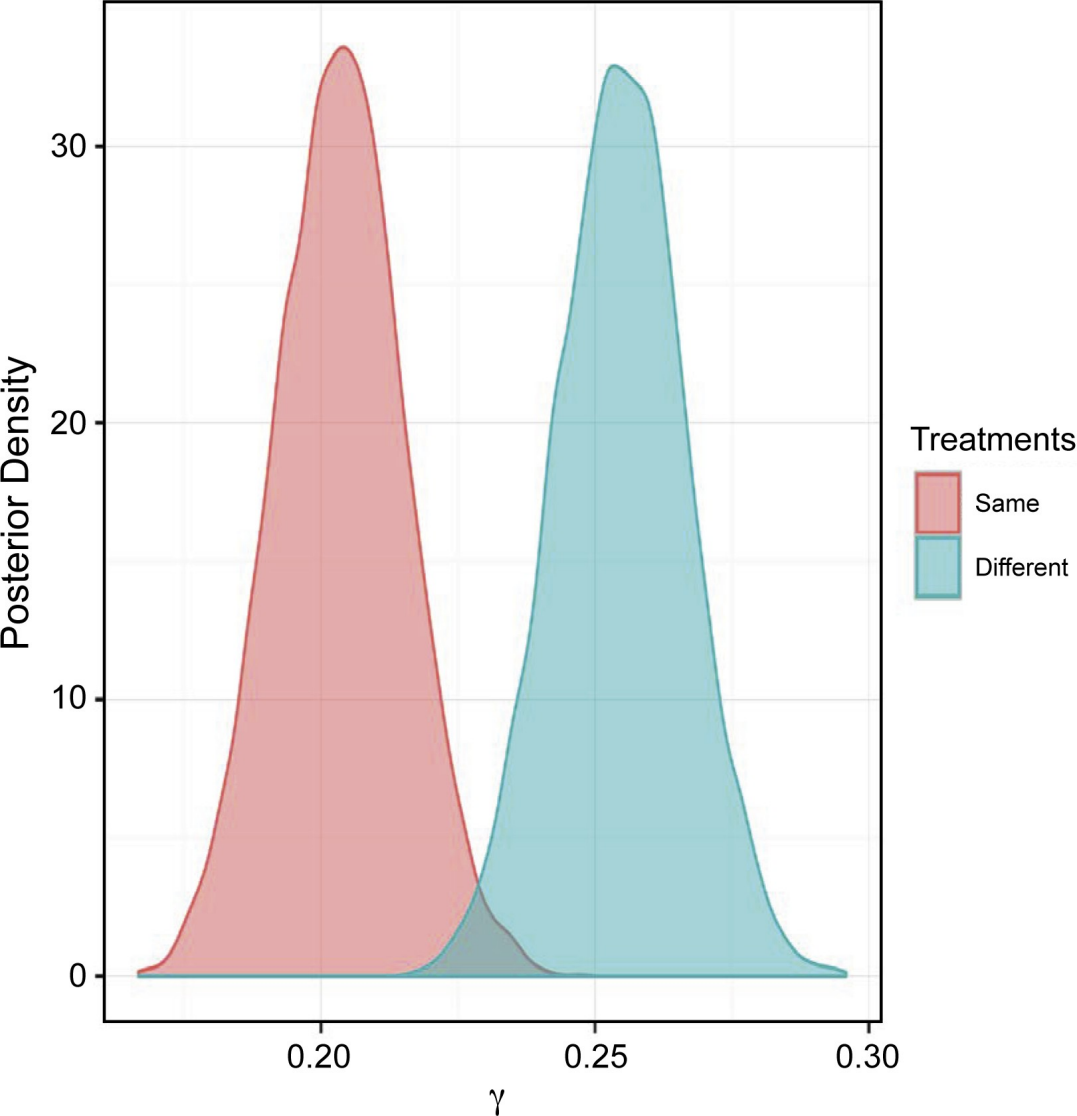
Posterior distributions of the probability that the isolate is resistant to multiple antimicrobials (i.e., γ_i_) stratified by the expected *in vitro* activity (i.e. bactericidal or bacteriostatic) of first and second treatment. Same = same *in vitro* effect on bacterial growth, i.e., bactericidal followed by bactericidal or bacteriostatic followed by bacteriostatic or different = different *in vitro* effect on bacterial growth, i.e., bactericidal followed by bacteriostatic or bacteriostatic followed by bactericidal.

However, when examining the posterior distribution of γ (where γ_i_ = number of resistant tests for an isolate), the posterior probability of γ_different_ > γ_same_ was 99% (**Fig 7 and Table 5**).

**Fig 8:**
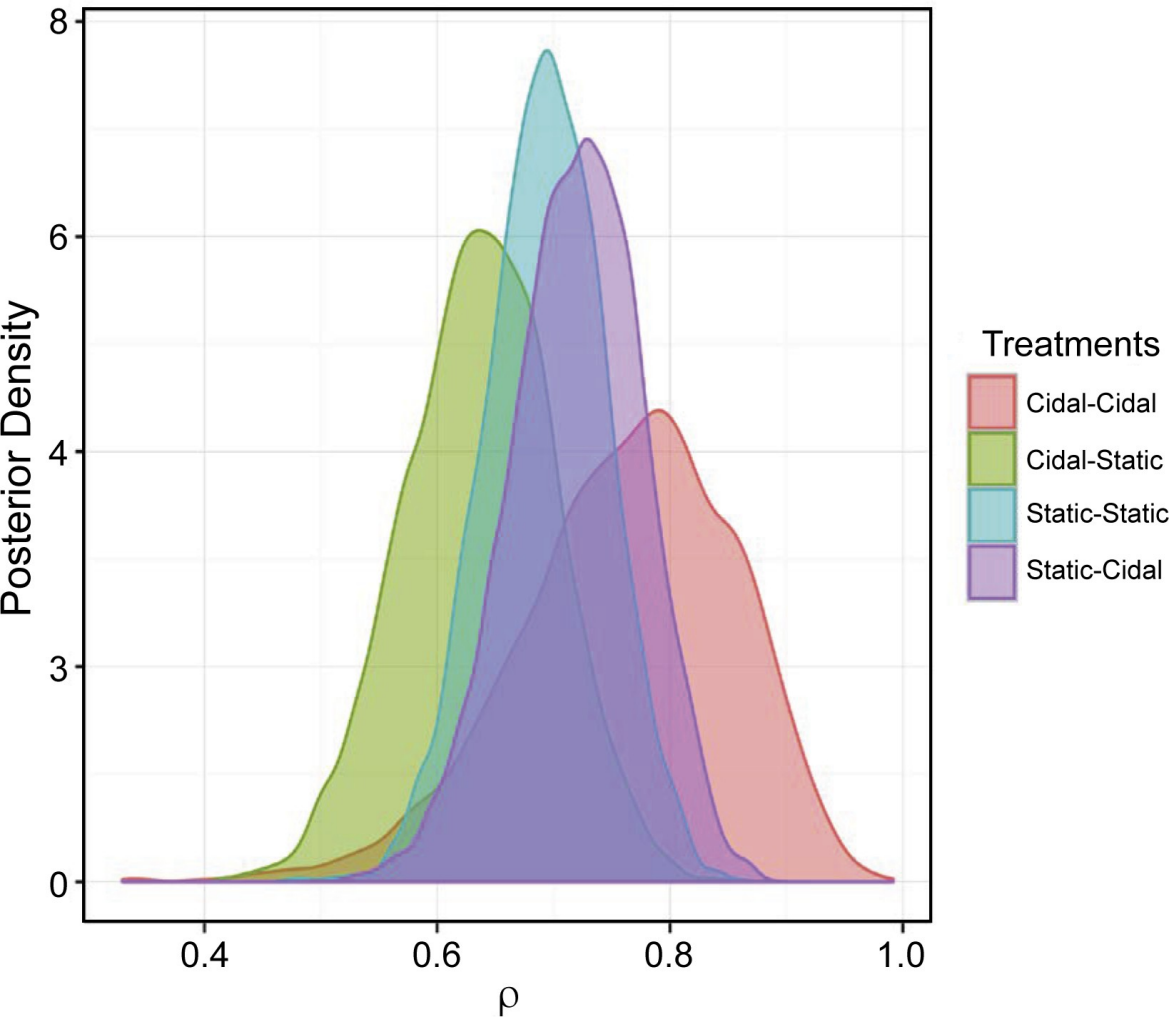
Posterior distribution of the probability that the isolate is resistant to at least one antimicrobial (i.e., ρ_i_) stratified by the expected *in vitro* activity (i.e. bactericidal or bacteriostatic) of first and second treatment. Cidal-Cidal = bactericidal first treatment followed by bactericidal retreatment; Cidal-Static = bactericidal first treatment followed by bacteriostatic retreatment; Static-Static = bacteriostatic first treatment followed by bacteriostatic retreatment; and Static-Cidal = bacteriostatic first treatment followed by bactericidal retreatment.

The results of the analysis from Objective 2 Model 1 suggest that the sequential administration of antimicrobial treatments with different effects on bacterial growth may be associated with higher numbers of resistant isolates and elevated MIC outcomes. Objective 2 Model 2 explores whether the sequence of bactericidal and bacteriostatic treatments has an impact on the probability of recovering a resistant BRD isolate. This analysis suggests that there is little impact of the treatment scheme sequence on the probability of identifying an isolate that is resistant to at least one antimicrobial (ρ). The specific posterior distributions and the 95% CI of ρ are shown in **Fig 8** and **Table 3**. Similarly, the posterior probability of an organism being resistant to at least one antimicrobial is presented in **Table 7**.

**Table 7:** Posterior probability that *p*_i+1_ is greater than p_i_ (*i* -4-level treatment mechanism sequence for objective 2 model 2).

	Bactericidal + bactericidal	Bactericidal + bacteriostatic	Bacteriostatic + bacteriostatic	Bacteriostatic + bactericidal
Bactericidal + bactericidal	NA	0.16	0.28	0.35
Bactericidal + bacteriostatic	--	NA	0.73	0.81
Bacteriostatic + bacteriostatic	-	-	NA	0.62
Bacteriostatic + bactericidal	-	-	-	NA

*i* is the 4-level treatment scheme sequence for Objective 2 Model 2.

As reported in **Table 7**, the probability of an organism being resistant to at least one antibiotic (ρ) was similar for the different treatment combinations. Specifically, in 62%, 81%, and 35% of cases, the probability of an organism being resistant to at least one antibiotic was higher if animals received a bacteriostatic antimicrobial for first treatment followed by a bactericidal antimicrobial for retreatment of BRD when compared to bacteriostatic-bacteriostatic, bactericidal-bacteriostatic, and bactericidal-bactericidal treatment, respectively.

**Fig 9:**
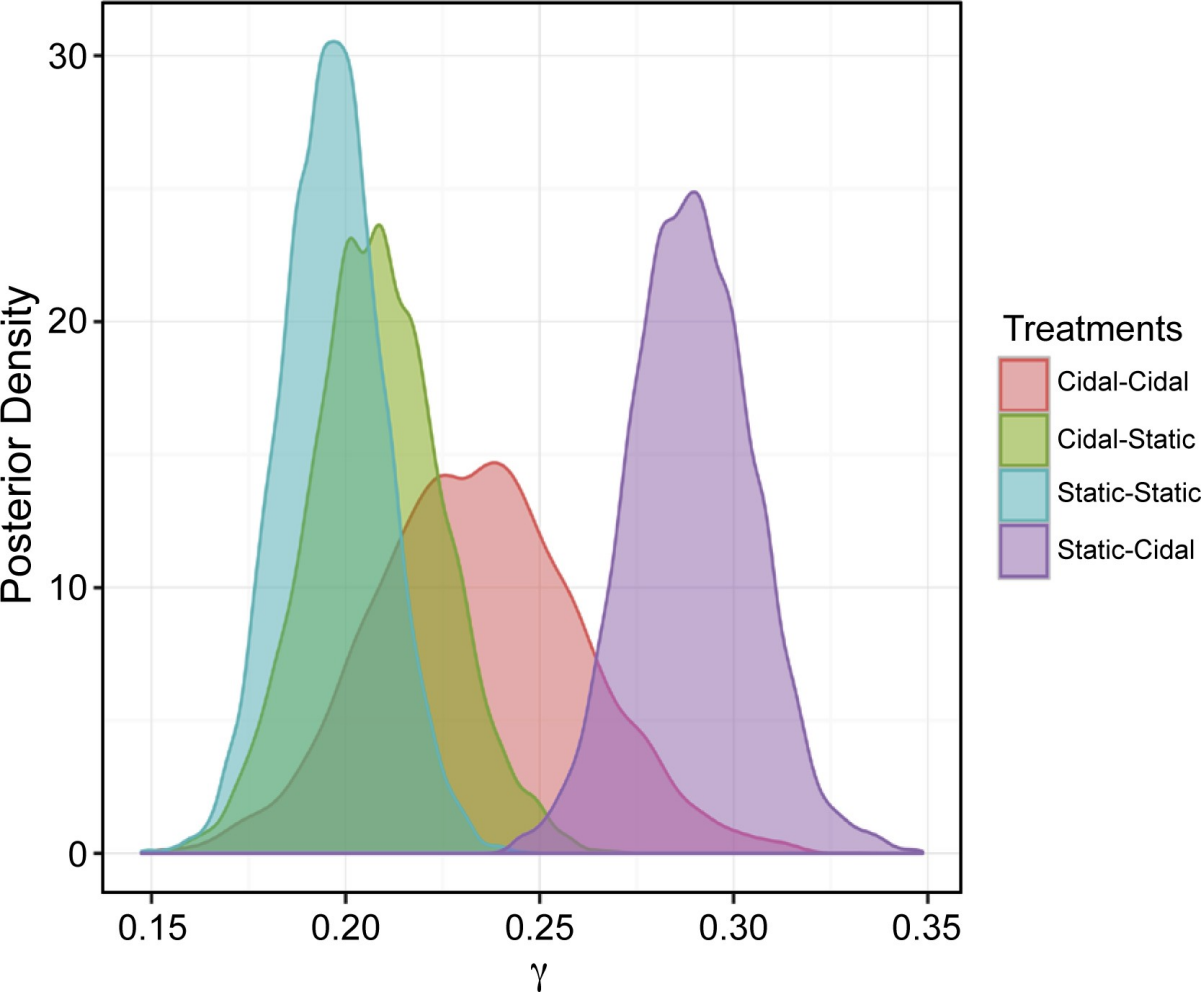
Posterior distributions of the probability that the isolate is resistant to multiple antimicrobials (i.e., γ_i_) stratified by the expected *in vitro* activity (i.e. bactericidal or bacteriostatic) of first and second treatment. Cidal-Cidal = bactericidal first treatment followed by bactericidal retreatment; Cidal-Static = bactericidal first treatment followed by bacteriostatic retreatment; Static-Static = bacteriostatic first treatment followed by bacteriostatic retreatment; and Static-Cidal = bacteriostatic first treatment followed by bactericidal retreatment.

With respect to the treatment, posterior gamma (γ) distributions shifted to the right in animals that received a first line bacteriostatic antimicrobial followed by retreatment with a bactericidal antimicrobial (**Fig 9**). This suggests that BRD pathogens isolated from these animals would be more likely to test resistant to more than one antimicrobial **(Table 5)**. A higher probability (>95%) of obtaining a resistant isolate from an animal receiving first-line bacteriostatic treatment followed by retreatment with a bactericidal antimicrobial than the other sequences was obtained(**Table 8**).

**Table 8:** Posterior probability that the γ_i_ is greater γ_i-1_ (*i* -4-level treatment mechanism sequence for objective 2 model 2) where γ_i_ = number of resistant tests for an isolate.

Objective 2 Model 2: Posterior distribution of 2-treatment sequence				
	bactericidal + bactericidal	bactericidal + bacteriostatic	bacteriostatic + bacteriostatic	bacteriostatic + bactericidal
bactericidal + bactericidal	NA	0.2	0.11	0.95
bactericidal + bacteriostatic	-	NA	0.32	0.99
bacteriostatic + bacteriostatic	-	-	NA	1
bacteriostatic + bactericidal	-	-	-	NA

*i* is the 4-level treatment mechanism sequence for Objective 2 Model 2.

## Discussion

In North America, BRD in feedlot cattle is associated with substantial economic losses due to the cost of treatment and deleterious effects on animal health and production [1–5]. Although BRD has a complex, multifactorial etiology, *M*. *haemolytica*, *P*. *multocida*, and *H*. *somni* are most often associated with clinical disease [16]. Therefore, use of antimicrobials is essential for the control and treatment of BRD in cattle [16]. Commonly used antimicrobial drugs that are approved in the US for treatment of BRD include ceftiofur, tilmicosin, tulathromycin, florfenicol, enrofloxacin, and danofloxacin [16]. In recent years, an increasing number of reports regarding the emergence of antimicrobial resistance among common BRD pathogens has been published [9, 17–23]. Typically, cattle affected with BRD are treated with a drug of a different antimicrobial class than the drug administered for first treatment or for disease prevention (metaphylaxis) [5]. Furthermore, when an animal does not respond to the first-line antimicrobial treatment or metaphylaxis, it may be retreated with one or more additional classes of antimicrobial drugs over subsequent days [16].

Data analyzed in the present study confirm that multiple antimicrobial classes are used to treat BRD (**Table 2**). In general, these antimicrobials either inhibit bacterial replication primarily through disruption of protein synthesis, or they cause bacterial death by inhibiting cell wall synthesis or disrupting DNA replication [16]. Regardless of the mechanism of action, antimicrobials exert a selection pressure on bacterial populations that may result in the emergence of antimicrobial resistant organisms. Specific factors that may contribute to resistance selection is the presence of antimicrobial concentrations that are below the MIC for the bacteria [24,25]. Bacterial pre-exposure to antimicrobials has also been implicated as a risk factor for AMR evolution during subsequent antimicrobial treatments [26–28]. Although previous laboratory studies have identified multidrug resistant (MDR) isolates from lung tissues collected from fatal BRD cases, antimicrobial resistance is not the only cause of death due to BRD [9,17,22,23]. Other factors such as stress, the timing of antimicrobial administration relative to the onset of the disease, a compromised host immune response, failure to achieve therapeutic drug concentrations at the site of infection, management factors such as poor nutrition, vaccination status or pre-existing or concurrent disease may also result in case fatalities.

Recently, the effect of sequential antimicrobial treatments on the development of antimicrobial resistance has been demonstrated for *Pseudomonas aeruginosa* and *Klebsiella pneumonia in vitro* [28,29]. In these laboratory studies the emergence of antimicrobial resistance also varied with the classes and concentrations of antimicrobials used for pre-exposure and sequential treatments. However, despite the frequent use of sequential treatments for the treatment of BRD in cattle, there are no data investigating if certain drug classes, doses, or drug regimens may promote, or minimize, the emergence of AMR when used sequentially.

To our knowledge, this survey is the first report that specifically considers AMR in livestock in the context of retreatments by different classes of antimicrobials (i.e., bacteriostatic and bactericidal) as well as different individual drugs (e.g., tulathromycin versus ceftiofur). Macrolides, such as tulathromycin and tildipirosin, are appealing as first-line treatments for the control of BRD (metaphylaxis) in high risk cattle due to their efficacy and long residence times in plasma and lung tissues. However, metaphylactic treatment with tulathromycin has been associated with a higher prevalence of multidrug resistant *M*. *haemolytica* shedding in cattle [17,30]. One explanation for the elevated antimicrobial resistance of *M*. *haemolytica* and *P*. *multocida* is the persistence of macrolides at concentrations that are below the MIC for the target organism [24,25,31]. During minimal inter-treatment intervals of 3–5 days, macrolides may still be present at biologically active concentrations that could allow for drug-drug interactions to occur when a second antimicrobial treatment is administered. Therefore, longer inter-treatment intervals are recommended when using antimicrobials with longer elimination half-lives [16]. Unfortunately the interval between treatments was not recorded on the submission histories analyzed for the present study, so the impact of post-treatment interval on the emergence of AMR could not be assessed. Even so, this report provides insights into potential critical control points for antimicrobial stewardship in livestock production systems.

The exploratory data presented in this report suggest that treatment protocols involving metaphylaxis, or first-line treatment, with a bacteriostatic antimicrobial followed by retreatment with a bactericidal antimicrobial may be associated with an increased frequency of resistant BRD pathogen isolation. This observation may be due to the fact that bacteriostatic activity may antagonize the effect of bactericidal drugs. Specifically, bactericidal drugs act on bacteria that are in a growth phase; thus, the inhibitory activity of a bacteriostatic drug on the replication of bacteria may result in diminished activity of a subsequent bactericidal treatment [11].

It is noteworthy that the classification of antimicrobials as bactericidal and bacteriostatic is based on the concentration required to sterilize bacterial cultures *in vitro*. As such, this system of classification may not be absolute for every combination of antimicrobial and bacterial isolate. For example, it has been reported in previous *in vitro* and *ex vivo* pharmacodynamics studies conducted against pneumonia pathogens of pigs and cattle that oxytetracycline and florfenicol may have bactericidal as opposed to bacteriostatic activity under certain conditions [32,33]. However, despite a few exceptions, broad mechanisms of action, such as protein synthesis inhibition and impacts on the growth of the bacterial cell wall, tend to be conserved within these mechanistic classes. Therefore, this classification serves as a useful guideline for clinicians in broadly consider the pharmacodynamics of the antimicrobial and to determine when two drugs may show synergistic or antagonistic interactions when used in combination [12].

Our data suggest that the number of treatments as well as alternating antimicrobial drug classes may impact antimicrobial resistance patterns. Damas *et al*. used three bactericidal antimicrobial classes (penicillins, cephalosporins, and fluoroquinolones) to treat serious infections in intensive care unit patients for 8-month periods over a 2-year study duration. After studying the effect of the sequential use of these three antimicrobial classes, antimicrobial rotation was associated with a higher risk for the development of antimicrobial resistance [34]. Similarly, in our study, potential for selection of antibiotic-resistant bacteria increased when more treatments were administered and when treatment exposure changed from one class to another antimicrobial class.

Specifically, the heat map (**Fig 2**) highlights how various antimicrobial combinations may influence the frequency of isolation of resistant *M*. *haemolytica*. For example, the combination of the bacteriostatic antimicrobial, tulathromycin, as the first-line treatment and the bactericidal antimicrobial, ceftiofur, as the second-line treatment increased the number of bacterial isolates that were resistant to danofloxacin (Fig 2A), enrofloxacin (Fig 2B), florfenicol (Fig 2C), spectinomycin (Fig 2D), tilmicosin (Fig 2E) and tulathromycin (Fig 2F). The use of tildipirosin as the first-line treatment and ceftiofur as the second-line treatment was also associated with an increased frequency of isolation of resistant *M*. *haemolytica*, specifically for florfenicol. This is not unexpected, given that tulathromycin and tildipirosin are in the same class of antimicrobials and have a similar mechanism of action. As previously mentioned, one explanation for this observation is that bactericidal antimicrobials, such as ceftiofur, that inhibit cell wall synthesis, are most effective against rapidly dividing bacteria. Previous exposure to bacteriostatic antimicrobials, such as tulathromycin, that inhibit bacterial replication may therefore reduce the number of actively dividing bacteria that are susceptible to the effects of ceftiofur resulting in drug antagonism.

Although class-switching is one potential explanation for this observation, the increase in the number of resistant isolates could also be attributed to this treatment combination being associated with drugs that are typically formulated with long durations of activity. These formulations may therefore represent a more significant selection pressure for resistant bacteria. However, the use of ceftiofur as the first-line treatment and tulathromycin as the second-line treatment resulted in recovery of fewer resistant *M*. *haemolytica* isolates. This observation suggests that the sequence of antimicrobial drug treatments, and not the duration of antimicrobial activity, contributed to the frequency of isolation of a resistant organism.

Differences between antimicrobial resistance patterns were examined by evaluating the MIC distributions of *M*. *haemolytica* for ceftiofur, danofloxacin, enrofloxacin, florfenicol, spectinomycin, tetracycline, tilmicosin, and tulathromycin (**Fig 1**). With the exception of ceftiofur and florfenicol, the number of resistant *M*. *haemolytica* isolates was greater when different antimicrobial classes were used for the first and second treatments. However, the number of susceptible *M*. *haemolytica* isolates did not differ with treatment drug class. In general, resistance to ceftiofur was rare, even in isolates obtained from animals treated with different antimicrobial classes. However, it is speculated that CLSI-approved breakpoints may not be accurate for ceftiofur against *M*. *haemolytica* and *P*. *multocida* for treatment of respiratory disease. It is believed that exposure to antimicrobials offers an advantage to resistant mutants in competition with the susceptible wild-type population [11]. However, the impact of multidrug combinations of different classes on positive selection of resistant mutants has not been closely examined. In a previous report, authors described a change in the ratio of doxycycline-resistant and doxycycline-sensitive *E*. *coli* following treatment with doxycycline alone or in combination with erythromycin [14]. The doxycycline-resistant mutants outnumbered the susceptible wild-type population of *E*.*coli* in both treatment conditions, but there was greater selection for the resistant mutants with the combination treatment. Van Loon *et al*. also reported that bacteria exhibit reduced susceptibility during treatment when different classes of antimicrobials were used [35].The results of the present survey are consistent with these reports given that they suggests that using a combination of different classes of antimicrobials may increase the risk of selection of resistant mutants.

Our ability to assess the impact of different drug classes on AMR for *P*. *multocida* and *H*. *somni* was limited in the present study due to the relatively small number of isolates with associated treatment histories that were available for analysis. It is known that the MIC distribution for *P*. *multocida* and *H*. *somni* may not have the same pattern as *M*. *haemolytica* isolates. In a previous report, pre-exposure to tulathromycin was associated with the development of bacterial resistance in *M*. *haemolytica* but not in *P*. *multocida* [36]. The number of *M*. *haemolytica* isolates compared to the number of *P*. *multocida* and *H*. *somni* isolates may influence the observations of this study. Although use of different mechanistic classes of antimicrobials was found to be associated with an increased frequency of isolation of resistant BRD pathogens, the relatively small number of *P*. *multocida* and *H*. *somni* isolates that were present in this dataset suggests that further investigation that specifically target these populations are needed before definitive and overarching conclusions can be made.

Although antimicrobial resistance has been a concern of scientists for decades, and specific BRD pathogen resistance was first reported over 40 years ago [37], our study appears to be the first investigation of the effects of treatment frequency and drug class selection on subsequent isolation of AMR organisms in cattle with BRD. The majority of published investigations have focused on the impact of exposure to a single class of antimicrobial on the emergence of antimicrobial resistance in feedlot cattle [17,21,30,38]. However, Kanwar and others reported that oral administration of chlortetracycline (CTC) in feed after treatment with ceftiofur crystalline free acid (CCFA) injection increased the recovery of ceftiofur-resistant bacteria from the feces of feedlot cattle [39]. Although this study suggested that a combination of bactericidal (CCFA) and bacteriostatic (CTC) antimicrobials may increase the risk of isolating AMR bacteria, they did not attempt to investigate the impact of treatment sequence or multiple antimicrobial classes on antimicrobial resistance outcomes.

One of the challenges with this study, and similar datasets [7, 9, 10], is that samples submitted to veterinary diagnostic laboratories may represent an inherently biased population of non-responding BRD cases that may not represent the population at large. As such, responding cattle are essentially excluded from the analysis. Furthermore, these retrospective studies assume that the treatment history and treatment sequence provided by the referring veterinarian is accurate. As such, concurrent use of oral antimicrobials in feed, for example, may not have been disclosed on the submission form and could potentially have increased the risk of selection of AMR organisms [39]. A further limitation is that data regarding certain antimicrobial combinations were sparse while others were more abundant. Specifically, the data were assembled before many of the recently approved long-acting antimicrobials for BRD, such as tildipirosin and gamithromycin, were in common use. Despite these limitations, the current study is hypothesis-generating and provides a novel approach for analyzing AMR microbes from BRD cases where multiple treatments with antimicrobial classes are evaluated.

The impact of multiple antimicrobial treatments represents an understudied area of research in veterinary medicine. It has been reported that feedlot cattle are commonly treated with antimicrobials more than once if the initial response is inadequate and that cattle that receive multiple antimicrobial treatments exhibit higher mortality rates from disease [5, 10]. Furthermore, animals that fail to respond to the initial treatment with one class of drug (e.g., bacteriostatic) are usually retreated with a different class of drug (e.g., bactericidal), which suggests a lack of consensus on any particular retreatment protocol [5, 16]. This lack of consensus is likely due to the scarcity of literature on pathogen response to multiple treatment regimens with different classes of antimicrobial agents. Our study suggests that sequential treatment with different classes of antimicrobials may be a risk factor for developing drug resistance. Therefore, a review of antimicrobial pre-exposure should be taken before the initiation of subsequent antimicrobial therapy to assist with mitigating the emergence of antimicrobial resistance in cattle infected with BRD.

As concern about the impact of AMR microbes on animal and public health increases, additional knowledge from studies such as the present survey is needed to investigate interventions that could potentially reduce the development of antimicrobial resistance. Furthermore, a microbiological diagnosis may be beneficial to assist with treatment selection for BRD of unknown etiology. Unfortunately, the time it takes to obtain AMR isolate results and the associated costs are two major limitations for the use of laboratory microbiology in veterinary medicine [40]. Furthermore, this study demonstrates the value and importance of including comprehensive treatment histories to accompany the submission of veterinary diagnostic laboratory samples. Taken together, studies of antimicrobial sensitivity patterns can serve as a guide to assist veterinarians in developing and implementing effective antimicrobial stewardship programs and protocols. Future studies on the relationship between treatment and antimicrobial resistance could assist practitioners in making decisions regarding antimicrobial retreatments when cattle exhibit BRD relapses.

These exploratory data suggest that treatment protocols stipulating first-line treatment with a bacteriostatic followed by second-line treatment with a bactericidal may increase the probability of emergence of drug resistance. As concern about antimicrobial resistance increases from an animal and public health perspective, this knowledge suggests potential ways to reduce the development of resistance. The hypothesis that the impact of an antimicrobial on bacterial growth may be associated with the risk of increased resistance needs to be tested in a clinical trial. Such a trial would also need to determine whether treatment efficacy is affected by a change in treatment protocol or post-treatment interval. If treatment effectiveness proves to be the same, then we may have an avenue by which to reduce the induction of resistance via the recommendation that veterinarians tailor their treatment regimens to reduce the potential for AMR development.

Although this study is hypothesis-generating, it has several strengths. The data set is reasonably large for the questions we asked. Although a great deal of data was missing, we limited our analysis to specific questions to avoid impact due to this missing data. Furthermore, we recognized the limits of the passively collected and hypothesis-generating nature of the data by not formally testing a hypothesis. The zero-inflated beta-binomial model that we used is an intuitive model that fit the underlying data well. We could not adjust this model for any confounders because of missing data; however, given the cross-sectional nature of the data, any attempt to adjust for confounders to improve causal inference would have been misleading and was thus avoided.

Our overall interpretation of the data suggests that there is direct correlation between the number of treatments to which an animal was exposed and the emergence of treatment resistance in samples submitted to a veterinary diagnostic laboratory for analysis. In addition, sequential treatments of BRD and the use of antimicrobials with different mechanisms of antibacterial activity (i.e., -static versus -cidal) may serve as a risk factor for the development of AMR.

## Supporting information

S1 Table A subset record of BRD cases submitted to the ISU-VDL for objective 1. (DOCX)

S2 Table A subset record of BRD cases submitted to the ISU-VDL for objective 2. (DOCX)

S3 Table Susceptibility criteria of antimicrobials against bacterial isolates obtained from BRD cases. (DOCX)
