# Supplementary material for: Association between antimicrobial drug class for treatment and retreatment of bovine respiratory disease (BRD) and frequency of resistant BRD pathogen isolation from veterinary diagnostic laboratory samples
Source: PLoS One. 2019 Dec 13;14(12):e0219104. doi: 10.1371/journal.pone.0219104 (PMC6910856; doi:10.1371/journal.pone.0219104)
Supplement: S1 Table — (DOCX) [file pone.0219104.s001.docx]

**Supplementary File**

**Table S1.** **A subset record of BRD cases submitted to the ISU-VDL for objective 1.**

| **Year** | **2013** | | | **2014** | | | **2015** | | | **Total** |
| --- | --- | --- | --- | --- | --- | --- | --- | --- | --- | --- |
| Accessions with 1 isolate | 165 | | | 184 | | | 139 | | | 488 |
| Accessions with 2 isolates | 28 | | | 51 | | | 54 | | | 133 |
| Accessions with 3 isolates | 0 | | | 4 | | | 5 | | | 9 |
| **Total Accessions** | 193 | | | 239 | | | 198 | | | 630 |
| **Organisms (Culture)** | **MH** | **PM** | **HS** | **MH** | **PM** | **HS** | **MH** | **PM** | **HS** | **Total** |
| Isolates from submissions with treatment history | 113 | 56 | 52 | 127 | 90 | 81 | 106 | 94 | 62 | 781 |
| **Total Isolates/ year** | 221 | | | 298 | | | 262 | | |  |
| **Owner Location (State)** | | | | | | | | | | |
| Missing | 11 | 5 | 4 | 15 | 8 | 10 | 8 | 14 | 8 | 83 |
| CO | 0 | 0 | 0 | 1 | 0 | 0 | 0 | 0 | 0 | 1 |
| FL | 9 | 2 | 1 | 2 | 3 | 2 | 0 | 0 | 0 | 19 |
| GA | 1 | 1 | 0 | 0 | 0 | 0 | 0 | 0 | 0 | 2 |
| IA | 70 | 39 | 38 | 83 | 68 | 54 | 68 | 64 | 44 | 528 |
| ID | 1 | 1 | 0 | 1 | 0 | 0 | 0 | 0 | 0 | 3 |
| IL | 4 | 0 | 1 | 2 | 0 | 1 | 6 | 2 | 2 | 18 |
| IN | 0 | 0 | 0 | 1 | 1 | 0 | 2 | 1 | 0 | 5 |
| MN | 3 | 6 | 4 | 7 | 5 | 3 | 8 | 9 | 4 | 49 |
| MO | 0 | 0 | 0 | 3 | 2 | 1 | 2 | 0 | 1 | 9 |
| NC | 0 | 0 | 0 | 0 | 0 | 0 | 1 | 0 | 0 | 1 |
| NE | 1 | 0 | 0 | 2 | 0 | 3 | 4 | 2 | 1 | 13 |
| OH | 1 | 0 | 0 | 2 | 1 | 1 | 0 | 0 | 0 | 5 |
| PA | 0 | 0 | 0 | 1 | 1 | 0 | 0 | 0 | 0 | 2 |
| SC | 1 | 0 | 0 | 0 | 0 | 0 | 0 | 0 | 0 | 1 |
| SD | 8 | 1 | 4 | 7 | 1 | 5 | 5 | 1 | 2 | 34 |
| TX | 2 | 1 | 0 | 0 | 0 | 0 | 0 | 0 | 0 | 3 |
| VT | 0 | 0 | 0 | 0 | 0 | 1 | 0 | 0 | 0 | 1 |
| WI | 0 | 0 | 0 | 0 | 0 | 0 | 1 | 1 | 0 | 2 |
| WV | 1 | 0 | 0 | 0 | 0 | 0 | 1 | 0 | 0 | 2 |
| **Facility Type** | | | | | | | | | | |
| Missing | 21 | 12 | 4 | 20 | 14 | 12 | 12 | 11 | 8 | 114 |
| Confinement | 35 | 22 | 22 | 42 | 24 | 20 | 20 | 16 | 6 | 207 |
| Dairy | 6 | 4 | 2 | 0 | 1 | 0 | 5 | 1 | 0 | 19 |
| Feedlot | 34 | 12 | 15 | 50 | 35 | 40 | 54 | 49 | 40 | 329 |
| Pasture | 17 | 6 | 9 | 15 | 16 | 9 | 15 | 17 | 8 | 112 |
| **Weight Range (kg)** | | | | | | | | | | |
| Missing | 10 | 9 | 8 | 15 | 8 | 6 | 12 | 6 | 9 | 83 |
| <100 kg | 22 | 15 | 12 | 15 | 11 | 7 | 18 | 16 | 11 | 127 |
| 101 - 200 kg | 16 | 13 | 14 | 22 | 29 | 30 | 17 | 31 | 21 | 193 |
| 201 - 300 kg | 40 | 9 | 15 | 50 | 24 | 27 | 33 | 29 | 14 | 241 |
| 301 - 400 kg | 14 | 6 | 3 | 14 | 5 | 9 | 10 | 7 | 7 | 75 |
| 401 - 500 kg | 5 | 2 | 0 | 10 | 9 | 2 | 6 | 3 | 0 | 37 |
| >500 kg | 6 | 2 | 0 | 1 | 4 | 0 | 10 | 2 | 0 | 25 |
| **Breed** |  |  |  |  |  |  |  |  |  |  |
| Missing | 11 | 5 | 5 | 15 | 9 | 7 | 14 | 10 | 9 | 85 |
| Dairy | 22 | 18 | 19 | 20 | 29 | 27 | 25 | 36 | 24 | 220 |
| Beef | 80 | 33 | 28 | 92 | 52 | 47 | 67 | 48 | 29 | 476 |
| **Sex** | | | | | | | | | | |
| Missing | 39 | 21 | 22 | 57 | 37 | 41 | 43 | 29 | 20 | 309 |
| Castrate | 22 | 10 | 12 | 27 | 25 | 22 | 16 | 29 | 20 | 183 |
| Female | 37 | 17 | 10 | 31 | 15 | 10 | 28 | 14 | 10 | 172 |
| Male | 15 | 8 | 8 | 12 | 13 | 8 | 19 | 22 | 12 | 117 |
| **Vaccination Status** | | | | | | | | | | |
| Missing | 22 | 10 | 11 | 28 | 21 | 13 | 26 | 26 | 11 | 168 |
| Yes | 87 | 39 | 35 | 93 | 66 | 63 | 75 | 60 | 47 | 565 |
| No | 4 | 7 | 6 | 6 | 3 | 5 | 5 | 8 | 4 | 48 |
| **PCR evidence of concurrent BRDC viral infection** | | | | | | | | | | |
| Missing | 6 | 4 | 4 | 12 | 9 | 11 | 9 | 7 | 7 | 69 |
| Positive | 36 | 17 | 16 | 50 | 29 | 26 | 40 | 31 | 17 | 262 |
| Negative | 71 | 35 | 32 | 65 | 52 | 44 | 57 | 56 | 38 | 450 |
| **PCR evidence of concurrent *Mycoplasma bovis* infection** | | | | | | | | | |  |
| Missing | 40 | 13 | 10 | 37 | 23 | 12 | 15 | 15 | 8 | 173 |
| Positive | 45 | 30 | 32 | 61 | 43 | 51 | 52 | 55 | 41 | 410 |
| Negative | 28 | 13 | 10 | 29 | 24 | 18 | 39 | 24 | 13 | 198 |
