# Supplementary material for: Association between antimicrobial drug class for treatment and retreatment of bovine respiratory disease (BRD) and frequency of resistant BRD pathogen isolation from veterinary diagnostic laboratory samples
Source: PLoS One. 2019 Dec 13;14(12):e0219104. doi: 10.1371/journal.pone.0219104 (PMC6910856; doi:10.1371/journal.pone.0219104)
Supplement: S2 Table — (DOCX) [file pone.0219104.s002.docx]

**Supplementary File**

**Table S2. A subset record of BRD cases submitted to the ISU-VDL for objective 2.**

| **Year** | **2013** | | | **2014** | | | **2015** | | | **Total** |
| --- | --- | --- | --- | --- | --- | --- | --- | --- | --- | --- |
| Accessions with 1 isolate | 53 | | | 49 | | | 35 | | | 137 |
| Accessions with 2 isolates | 9 | | | 12 | | | 10 | | | 31 |
| Accessions with 3 isolates | 0 | | | 3 | | | 1 | | | 4 |
| **Total Accessions** | 62 | | | 64 | | | 46 | | | 172 |
| **Organisms (Culture)** | **MH** | **PM** | **HS** | **MH** | **PM** | **HS** | **MH** | **PM** | **HS** | **Total** |
| Isolates with first and second treatment history | 39 | 17 | 15 | 39 | 18 | 25 | 23 | 25 | 10 | 211 |
| **Total Isolates/ year** | 71 | | | 82 | | | 58 | | |  |
|  |  | | |  | | |  | | |  |
| **Owner Location (State)** | | | | | | | | | | |
| Missing | 2 | 2 | 0 | 9 | 2 | 2 | 4 | 3 | 0 | 24 |
| FL | 7 | 1 | 0 | 0 | 0 | 0 | 0 | 0 | 0 | 8 |
| IA | 22 | 10 | 11 | 24 | 13 | 21 | 14 | 18 | 8 | 141 |
| IL | 3 | 0 | 0 | 1 | 0 | 0 | 0 | 0 | 0 | 4 |
| IN | 0 | 0 | 0 | 1 | 1 | 0 | 0 | 0 | 0 | 2 |
| MN | 2 | 3 | 2 | 1 | 1 | 1 | 3 | 3 | 1 | 17 |
| MO | 0 | 0 | 0 | 2 | 0 | 0 | 0 | 0 | 0 | 2 |
| NE | 1 | 0 | 0 | 0 | 0 | 0 | 1 | 1 | 1 | 4 |
| PA | 0 | 0 | 0 | 1 | 1 | 0 | 0 | 0 | 0 | 2 |
| SD | 2 | 1 | 2 | 0 | 0 | 1 | 1 | 0 | 0 | 7 |
| **Facility Type** | | | | | | | | | | |
| Missing | 8 | 4 | 2 | 5 | 2 | 1 | 3 | 2 | 0 | 27 |
| Confinement | 10 | 9 | 8 | 16 | 6 | 7 | 5 | 5 | 0 | 66 |
| Dairy | 1 | 1 | 2 | 0 | 0 | 0 | 0 | 0 | 0 | 4 |
| Feedlot | 12 | 3 | 1 | 17 | 10 | 16 | 13 | 14 | 7 | 93 |
| Pasture | 8 | 0 | 2 | 1 | 0 | 1 | 2 | 4 | 3 | 21 |
| **Weight Range (kg)** | | | | | | | | | | |
| Missing | 1 | 2 | 3 | 3 | 1 | 0 | 1 | 2 | 0 | 13 |
| <100 kg | 6 | 4 | 4 | 5 | 3 | 3 | 4 | 5 | 3 | 37 |
| 101 - 200 kg | 9 | 6 | 3 | 8 | 7 | 11 | 3 | 7 | 3 | 57 |
| 201 - 300 kg | 17 | 3 | 5 | 19 | 4 | 7 | 11 | 8 | 2 | 76 |
| 301 - 400 kg | 2 | 0 | 0 | 2 | 1 | 3 | 2 | 3 | 2 | 15 |
| 401 - 500 kg | 2 | 2 | 0 | 2 | 1 | 1 | 0 | 0 | 0 | 8 |
| >500 kg | 2 | 0 | 0 | 0 | 1 | 0 | 2 | 0 | 0 | 5 |
| **Breed** |  |  |  |  |  |  |  |  |  |  |
| Missing | 6 | 3 | 0 | 4 | 1 | 0 | 2 | 3 | 2 | 21 |
| Dairy | 5 | 7 | 8 | 10 | 9 | 11 | 6 | 14 | 3 | 73 |
| Beef | 28 | 7 | 7 | 25 | 8 | 14 | 15 | 8 | 5 | 117 |
| **Sex** | | | | | | | | | | |
| Missing | 12 | 7 | 4 | 19 | 8 | 11 | 9 | 9 | 1 | 80 |
| Castrate | 9 | 2 | 4 | 9 | 5 | 9 | 3 | 6 | 3 | 50 |
| Female | 14 | 6 | 5 | 8 | 3 | 1 | 5 | 2 | 4 | 48 |
| Male | 4 | 2 | 2 | 3 | 2 | 4 | 6 | 8 | 2 | 33 |
| **Vaccination Status** | | | | | | | | | | |
| Missing | 9 | 3 | 2 | 7 | 3 | 2 | 5 | 6 | 1 | 38 |
| Yes | 30 | 13 | 11 | 32 | 15 | 22 | 18 | 19 | 8 | 168 |
| No | 0 | 1 | 2 | 0 | 0 | 1 | 0 | 0 | 1 | 5 |
| **PCR evidence of concurrent BRDC viral infection** | | | | | | | | | | |
| Missing | 3 | 1 | 1 | 3 | 2 | 3 | 0 | 2 | 0 | 15 |
| Positive | 15 | 6 | 5 | 14 | 5 | 4 | 11 | 10 | 3 | 73 |
| Negative | 21 | 10 | 9 | 22 | 11 | 18 | 12 | 13 | 7 | 123 |
| **PCR evidence of concurrent *Mycoplasma bovis* infection** | | | | | | | | | | |
| Missing | 14 | 3 | 2 | 11 | 2 | 3 | 1 | 3 | 0 | 39 |
| Positive | 18 | 12 | 13 | 22 | 9 | 18 | 16 | 15 | 7 | 130 |
| Negative | 7 | 2 | 0 | 6 | 7 | 4 | 6 | 7 | 3 | 42 |
