# Supplementary material for: Association between antimicrobial drug class for treatment and retreatment of bovine respiratory disease (BRD) and frequency of resistant BRD pathogen isolation from veterinary diagnostic laboratory samples
Source: PLoS One. 2019 Dec 13;14(12):e0219104. doi: 10.1371/journal.pone.0219104 (PMC6910856; doi:10.1371/journal.pone.0219104)
Supplement: S3 Table — (DOCX) [file pone.0219104.s003.docx]

**Supplementary File**

**Table S3.** **Susceptibility criteria of antimicrobials against bacterial isolates obtained from BRD cases.**

| **Antimicrobial Tested** | **Interpretive Criteria** | **CLSI Validated Breakpoint (µg/mL)** | **Number of isolates** | | | **Total** |
| --- | --- | --- | --- | --- | --- | --- |
|  |  |  | ***M. haemolytica*** | ***P. multocida*** | ***H. somni*** |  |
| Ceftiofur | Susceptible | ≤ 2 | 101 | 60 | 50 | 211 |
|  | Intermediate | 4 | 0 | 0 | 0 | 0 |
|  | Resistant | ≥ 8 | 0 | 0 | 0 | 0 |
|  | No Interpretation | | 0 | 0 | 0 | 0 |
| Danofloxacin^1^ | Susceptible | ≤ 0.25 | 40 | 48 | 35 | 123 |
|  | Intermediate | 0.5 | 2 | 4 | 0 | 6 |
|  | Resistant | ≥ 1 | 59 | 8 | 15 | 82 |
|  | No Interpretation | |  |  |  |  |
| Enrofloxacin | Susceptible | ≤ 0.25 | 40 | 51 | 35 | 126 |
|  | Intermediate | 0.5 - 1 | 4 | 5 | 11 | 20 |
|  | Resistant | ≥ 2 | 57 | 4 | 4 | 65 |
|  | No Interpretation | |  |  |  | 0 |
| Florfenicol | Susceptible | ≤ 2 | 66 | 55 | 47 | 168 |
|  | Intermediate | 4 | 0 | 1 | 3 | 4 |
|  | Resistant | ≥8 | 35 | 4 | 0 | 39 |
|  | No Interpretation | |  |  |  | 0 |
| Tetracycline^2^ | Susceptible | ≤ 2 | 28 | 25 | 15 | 68 |
|  | Intermediate | 4 | 0 | 3 | 5 | 8 |
|  | Resistant | ≥8 | 73 | 32 | 30 | 135 |
|  | No Interpretation | |  |  |  | 0 |
| Spectinomycin | Susceptible | ≤ 32 | 45 | 40 | 22 | 107 |
|  | Intermediate | 64 | 1 | 1 | 6 | 8 |
|  | Resistant | ≥128 | 55 | 19 | 22 | 96 |
|  | No Interpretation | |  |  |  | 0 |
| Tilmicosin | Susceptible | ≤ 8 | 32 | 31 | 36 | 99 |
|  | Intermediate | 16 | 8 | 3 | 0 | 11 |
|  | Resistant | ≥ 32 | 61 | 26 | 14 | 101 |
|  | No Interpretation | |  |  |  | 0 |
| Tulathromycin | Susceptible | ≤ 16 | 41 | 41 | 29 | 111 |
|  | Intermediate | 32 | 3 | 6 | 6 | 15 |
|  | Resistant | ≥64 | 52 | 13 | 13 | 78 |
|  | No Interpretation | | 5 | 0 | 2 | 7 |

^1^ Sweeney M.T., Papich M.G., Watts J.L. 2017. New interpretive criteria for danofloxacin antibacterial susceptibility testing against Mannheimia haemolytica and Pasteurella multocida associated with bovine respiratory disease. *J Vet Diagn Invest*; 29(2):224-227

^2^ “Generic” breakpoints determined on the basis of published pharmacokinetic parameters in the designated species in combination with available target pathogen susceptibility data.
